# The Impact of the COVID-19 Pandemic on Affect, Fear, and Personality of Primary School Children Measured During the Second Wave of Infections in 2020

**DOI:** 10.3389/fpsyt.2021.803270

**Published:** 2022-01-17

**Authors:** Alessio Matiz, Franco Fabbro, Andrea Paschetto, Cosimo Urgesi, Enrica Ciucci, Andrea Baroncelli, Cristiano Crescentini

**Affiliations:** ^1^Department of Languages and Literatures, Communication, Education and Society, University of Udine, Udine, Italy; ^2^Department of Psychology, University of Rome La Sapienza, Rome, Italy; ^3^Institute of Mechanical Intelligence, Scuola Superiore Sant'Anna di Pisa, Pisa, Italy; ^4^Scientific Institute, IRCCS E. Medea, Neuropsychiatry and Neurorehabilitation Unit, Lecco, Italy; ^5^Department of Education, Languages, Intercultures, Literatures and Psychology, University of Florence, Florence, Italy

**Keywords:** children, personality, affect, COVID-19, mental health, anxiety, depression, spirituality

## Abstract

In relation to the COVID-19 pandemic outbreak, a large body of research has identified a negative impact on individuals' affectivity, frequently documented by increased prevalence of anxiety and depression symptoms. For children, this research was less extensive, was mainly based on caregivers' reports and neglected personality assessment. In order to measure the impact of the pandemic, and the fears it caused, on primary school children's affect and personality, 323 (180 boys and 143 girls) Italian third, fourth and fifth graders were assessed between October and November 2020, namely during the second wave of COVID-19 infections in Italy, with validated self-reports of affect (Positive and Negative Affect Scale for Children, PANAS-C), fear of COVID-19 (Fear of COVID-19 Scale, FCV-19S) and personality (junior Temperament and Character Inventory, jTCI). In comparison with PANAS-C and jTCI normative scores collected prior to the pandemic, data obtained from children in 2020 showed unchanged affect scores in the overall sample, a decrease of Positive Affect in girls, and a decrease in the Harm Avoidance and an increase in the Self-Transcendence scales of personality. Fear of COVID-19 scores were positively correlated with Negative Affect scores and negatively predicted by children's personality profile of resilience (calculated using scores on the Harm Avoidance and the Self-Directedness scales of personality). These results suggested that Italian primary school children, especially boys, maintained their pre-pandemic levels of affect (or restored them after the first COVID-19 wave) and partially diverged from the typical development of personality in an apparently positive sense, namely toward more courageous/optimistic and spiritual profiles. This sort of children's post-traumatic growth might also be attributed to children's family and education systems, which should continue to be supported to promote and maintain community mental health.

## Introduction

After the outbreak of the worldwide COVID-19 pandemic in early 2020 and the consequent public health policies put in action to contain the waves of infections, a large body of research has documented a worsening of public mental health. Various systematic reviews and meta-analyses reported increased emotional distress and increased risk for psychiatric disorders among the adult general population during 2020 (1–9). Less research has explored the impact of the pandemic emergency on the emotional well-being of children; the relevant reviews though resulted in reporting a negative psychological impact related to COVID-19 (10–16). Although the risk of death from COVID-19 is negligible for children and adolescents, they can nevertheless be as susceptible as adults to the psychological impact of the pandemic and its response measures (e.g., obligation to stay at home, interruption of both regular school and extracurricular activities attendance, physical distancing).

Most of the studies included in the above-mentioned reviews found that individuals' levels of anxiety and depression were the most frequent indicator of psychological distress, both in adults and children. Anxiety and depression are two forms of human suffering which have distinct and overlapping features. According to the model of Clark and Watson (17), they may share a component of general emotional distress, which can be labeled as negative affect (NA), and are differentiated by the levels of positive affect (PA), which is characteristically lower in depression than in anxiety. This model, together with the resulting scale for measuring positive and negative affectivity (i.e., the Positive and Negative Affect Scale, PANAS) (18), has been largely used both on adults and younger people (19–23). Positive and negative affect, considered as the set of transient and enduring evaluative feelings experienced by a person in response to salient events/conditions (24), can be therefore regarded as critical markers of the psychological condition of persons also in respect to the impact of the COVID-19 related crisis. It seems therefore particularly important to assess the levels of positive and negative affect in the population during the COVID-19 pandemic and to compare them with the normative levels collected before the pandemic. This pre- vs. during pandemic comparison, which has been performed for measures such as anxiety, depression and psychological well-being [e.g., (25–27)], has not been frequently carried out so far on affect scores. A study on a thousand full-time adult workers during the early stages of the pandemic in Germany revealed that their positive and negative affectivity did not change between December 2019 and March 2020, but decreased between March and May 2020 (28). A smaller study on adolescents (*n* = 34) and their parents (*n* = 67) conducted in the Netherlands revealed that adolescents' positive or negative affectivity did not change between 2018–19 and March 2020, while parents significantly reported a more negative affect in March 2020 in comparison to 2018–19 (29). The only existing study that assessed positive and negative affect of children (*n* = 34) during the pandemic (April-July 2020) and compared these scores with data collected prior to the pandemic (*n* = 101) did not find any difference in affect scores (30). The scarce information on this important aspect of individuals' mental health during the pandemic, in particular for children, urgently calls for a wider investigation.

In relation to negative affectivity, a salient emotion experienced by many persons during the pandemic is the fear of COVID-19. A self-report measure on this feeling was indeed developed in early 2020, the Fear of COVID-19 Scale (FCV-19S) (31). In this tool, for which factor analyses generally indicated a unidimensional structure, people are asked to evaluate both the physical and mental components of their fear of COVID-19. The FCV-19S has been extensively used since its introduction and made it possible to estimate the distribution of scores in separate samples (32), compare scores between samples of different countries (32, 33), and compare scores of a same population obtained in different time points (e.g., during the first vs. second wave of COVID-19) (34). The FCV-19S was originally developed for adults, but it was also employed in youth samples, in particular in adolescents (35–37). Nevertheless, given its small number of items, the relatively simple form of its statements and of the 5-point response scale in which respondents indicate their level of agreement with the statements, the FCV-19S was also administered to children as small as 7 years of age (38). Similarly to what is done in adults, it would thus be useful to further explore the depth and prevalence of fear of COVID-19 using the FCV-19S in children of different countries and during different phases of the pandemic, with the aim of providing children with the best environmental and psychological support in relation to this specific emotional sequelae of the pandemic.

An overarching aspect taken in consideration in many studies on the affective repercussion of the pandemic crisis is personality. Most of these studies assessed individuals' personality traits in combination with other measures, with the aim to link specific traits to various outcomes of interest, such as the level of distress, the way of perceiving the emergency, the form of behavioral adjustments to the emergency, and the degree of compliance to safety rules [e.g., (39–44)]. These studies were all carried out on adult samples. Although adults' personality is relatively stable, referring to “individual differences in characteristic patterns of thinking, feeling and behaving” (45), a number of researches have investigated whether the pandemic crisis has come to significantly change the overall personality profile of the population.

Studies on healthy adults' self-reports of personality collected during the pandemic did not gave a definite answer to this question: most of the studies found out that the scores collected during the pandemic with instruments such as the Brief HEXACO Inventory (46), the International Personality Item Pool's IPIP-NEO (47), the reduced Temperament and Character Inventory (48), the Personality Inventory for the DSM-5–Brief Form (49), or the various versions of the Big Five Inventory (50–53) remained stable (i.e., remained within one standard deviation of the normative means) in comparison with those collected before it [e.g., (39, 54–57)]. Other studies, however, found that scores changed beyond one standard deviation from the normative means [e.g., (58, 59)], or found significant changes in the pre- vs. during pandemic comparisons of scores: for example, significant changes were observed, using the Big Five Inventory-2 questionnaire (53), in the neuroticism and extraversion traits of the big-5 model of personality in a sample of 2,137 U.S. citizens who were tested before (early February 2020) and during (second half of March 2020) the pandemic outbreak in the U.S. (60). In yet another study, significant changes were observed in all the big-5 traits of personality in 480 alleged healthcare workers when using linguistic analyses of their social media data collected before (February 2020) and during (between February and April 2020) the pandemic (61).

Childhood is an important period of life for the development of an individual's personality, because in this period the interaction between individuals' inborn traits and their personal life events increasingly organizes the course of children's action, emotion and cognition and their subsequent personality development (62). The personality of children may therefore face important developmental changes: however, the evaluation of the psychological impact of the COVID-19 related crisis on children's personality can be performed by detecting possible changes in the typical development of personality. Such changes can be monitored, for example, on the basis of age-appropriate normative scores (collected prior to the pandemic) of instruments for personality assessment such as the Big Five Questionnaire for Children (63) or the junior Temperament and Character Inventory (64). Yet, the question of whether the psychological impact of the COVID-19 related crisis may have changed typical personality development of children has not been answered so far. It would also be useful to replicate in children the studies that highlighted which personality dimensions were associated with the health outcomes previously investigated in adults such as well-being and anxiety/depression.

Moreover, as evidenced in many studies on adults (44, 65–68), even for children a key factor impacting the individual ability to cope with the distress caused by the pandemic could be linked to the personality aspect of resilience. More in particular, in the seven-dimension model of personality measured by the Temperament and Character Inventory (TCI) (69) or its junior version (jTCI) (64), high and low resilience profiles can be effectively measured by focusing on the two dimensions of harm-avoidance (a temperamental trait reflecting the tendency to avoid behaviors due to intense response to aversive stimuli expressed as fear of uncertainty, quick fatigability, shyness of strangers, and pessimistic worry) (70) and self-directedness (a character trait referring to self-determination, self-acceptance, responsibility and reliability, and to being able to control, regulate, and adapt behavior in accordance to one's own goals and values) (70), which are respectively negatively and positively related to resilience (71–73). Thus, besides considering personality for either trying to monitor its possible changes after the pandemic or for investigating its general association with the affective impact of the pandemic, focusing on children's resilience profiles may also help explaining in a more specific way why the COVID-19 related crisis has affectively impacted some individuals differently from others.

In sum, in our study data on positive and negative affect, fear of COVID-19 and personality were collected in a sample of Italian primary school children. All data were collected through children's self-reports while they were at school. For affect, the Positive and Negative Affect Scale for Children (PANAS-C) (74, 75) was used, for the fear of COVID-19 the Fear of COVID-19 Scale (FCV-19S) (31, 76) and, for personality, the junior Temperament and Character Inventory (jTCI) (64, 77). Assessment was carried out between October and November 2020, during the second wave of the pandemic in Italy. The main aim of the study was (i) to compare normative PANAS and jTCI data (collected on independent samples of children before the pandemic) with data obtained during the pandemic period. In particular, in the period of assessment, Italian children had just returned to school after more than 6 months of school closure and the country was facing the ascending phase of the second pandemic wave without certainties about the degree of its sanitary, economic and social impact. The secondary aim of the study was (ii) to assess the levels of fear of COVID-19 in these same children and to link their levels of fear, positive affect and negative affect with their personality characteristics. This was done first by correlating the PANAS-C and FCV-19S with the jTCI scores and then by assessing the differences in PANAS-C and FCV-19S scores in two separate children groups: one with a low-resilience personality profile and the other with high-resilience. In this way, the present study tried to address some relevant questions that have partially or completely been overlooked in the literature so far: were affect and personality profiles of primary school children assessed during the second wave of the pandemic different from those collected in age-matched children before the pandemic? How were the primary school children's personality characteristics in 2020 related to children's levels of fear of COVID-19, positive affect and negative affect, also considering the aspect of high and low resilience?

## Materials and Methods

### Participants

Twenty-one classes from 14 primary schools of the North-East part of Italy (Friuli-Venezia Giulia region) participated in the assessment of the present study, as the initial stage of a successive attentional and self-regulation training program. A total of 361 third, fourth, and fifth graders were assessed. After excluding the data from 38 children (18 questionnaires were not complete, three questionnaires had been completed by children with intellectual disabilities, 17 jTCI reports had no valid responses for control items), the final sample consisted of 323 children (grade: 103 third, 75 fourth, 145 fifth; sex: 180 boys, 143 girls).

### Measures

#### Affect

Positive and negative affect were measured with the Italian version of the Positive and Negative Affect Scale for Children (PANAS-C) (74, 75). This tool was originally developed and validated on 9- to 12-year-old children, but it was also used for third graders [e.g., (78, 79)]. It is the child version of PANAS, the most frequently used scale to assess positive (PA) and negative affect (NA) in adults (18). In PANAS-C, respondents are asked to rate on a 5-point Likert scale (ranging from 1 = never to 5 = always) how often during the last weeks they have experienced each of the 30 positive or negative listed moods that in the tool are expressed by adjectives or very short expressions. In the Italian version, PA score is the sum of scores for 11 items and NA score is the sum of scores for 13 items. Example items are “Active” (for PA) and “Afraid” (for NA). Both the original and the Italian validation of PANAS-C showed two clearly differentiated factors (PA and NA) and good internal consistency reliability (alpha ≥ 0.85). For data collected for the present study in 2020, Cronbach's alphas were: 0.84 for PA and 0.87 for NA.

#### Personality

Personality was assessed with the Italian version of the junior Temperament and Character Inventory (jTCI) (64, 77). This is the child version of the widely known TCI personality inventory (69). It was developed and validated on 9- to 12-year-old children, and consists of 108 true/false items. Respondents are asked to express their general concordance/discordance with each statement. The jTCI has four temperament scales (Novelty Seeking, NS; Harm Avoidance, HA; Reward Dependency, RD; Persistence, P) and three character scales (Self-Directedness, SD; Cooperativeness, C; Self-Transcendence, ST). Temperament scales model the inborn neurobiological tendencies toward early emotions and the resultant behavioral reactions to distinct environmental stimuli. Character scales model, at the intra-, inter- and trans-personal level of the individual, the result of the interaction between temperament traits, socio-cultural influences, life events and intentional training. Example items are: “I get tense and worried in unfamiliar situations” (HA), “I often try new things for fun or thrills” (NS), “I don't open up much even with friends” (RD), “I work long after others give up” (P), “I feel strong enough, to master everything somehow” (SD), “I take good care not to hurt somebody with my actions” (C), “I believe in a higher force connecting all living beings” (ST). Cronbach's alphas for data collected for the present study in 2020 were: 0.63 for NS (18 items), 0.74 for HA (22 items), 0.47 for RD (nine items), 0.35 for P (six items), 0.65 for SD (20 items), 0.65 for C (20 items), 0.49 for ST (10 items).

#### Fear of COVID-19

The fear of COVID-19 was measured with the Italian version of the Fear of COVID-19 Scale (FCV-19S) (31, 76). This tool consists of seven items with a five-point rating scale (ranging from 1 = strongly disagree to 5 = strongly agree) and was developed for adults; nonetheless, it has been used in children as young as 7 years old (36–38). Example items are “I am very afraid of the coronavirus-19” and “I cannot sleep because I'm worrying about getting (or having) coronavirus-19”. As FCV-19S is recognized as an uni-dimensional measure (32), a total score is provided, with higher scores corresponding to greater fear of COVID-19. The FCV-19S showed good internal consistency (seven items; Cronbach's alpha = 0.80) when applied to children in our study. This was consistent for the different grades (alpha = 0.82 for third graders, alpha = 0.76 for fourth graders, alpha = 0.79 for fifth graders). Results of a confirmatory factor analysis using diagonally weighted least squares method on data of our study [χ^2^(14) = 32.3, *p* < 0.01; Root Mean Square Error of Approximation (90% Confidence Interval) = 0.06 (0.03;0.09), Comparative Fit Index = 0.99, Standardized Root Mean square Residual = 0.07] revealed an acceptable fit for the seven-item single-factor construct (80).

### Procedure

The study was carried out between October 13, 2020 and November 6, 2020. In this period Italy was experiencing the second wave of COVID-19 infections, which peaked on November 13, 2020 with 40,902 new daily cases and 550 daily deaths (81). In the Friuli-Venezia Giulia region, where the study took place, the restrictions applied in the initial weeks of the study (until November 6, 2020) were: compulsory face masks in public areas, distance learning in high schools and universities, no service after 12 a.m. for bars serving food and restaurants. After November 6, tighter restrictions were introduced: stay-home mandate between 10 p.m. and 5 a.m., closure of shopping malls during weekends and holidays, 50% capacity reduction on public transport, closure of indoor recreational and cultural venues, closure of indoor gyms, pools and leisure venues, and prohibition of non-professional contact sports (82). People had been informed by mass media that the pandemic was going to get worse before it got better.

Paper questionnaires were administered by school instructors to their pupils during teacher-led classes. The teachers had been previously instructed by researchers, during an online group meeting, in the procedure to be followed for administering the questionnaires: they had to read the instructions of each questionnaire to the class, explain any word/expression that the children had asked to clarify and refrain from suggesting any response to their students during the filling of the questionnaires.

Parents of all participants provided written informed consent for their children's inclusion in the study. The study was approved by the Ethics Committee of the University of Udine and all procedures performed in the study were in accordance with the ethical standards of the 1964 Helsinki declaration and its later amendments. Finally, all data were analyzed anonymously and data confidentiality was ensured.

### Statistical Analysis

Data analysis was conducted using R, version 3.6.3 (83). Power analysis was performed with GPower, version 3.1 (84). Missing values in participants' responses were found to be <2% and were imputed with the mean score of the whole sample for the corresponding item.

Primary analysis involved (i) testing the difference between the distribution of the PANAS-C and jTCI data obtained in October-November 2020 and the distribution of data from the PANAS-C and jTCI datasets obtained during the validation of these questionnaires in Italy (74, 76). The difference was tested using robust independent samples *t*-tests separately for boys, girls, and boys and girls together. Bonferroni correction for multiple comparisons was applied in each separate group.

The PANAS-C validation dataset included data of fourth and fifth graders collected in 2014 (*n* = 331, 51.7% boys). The jTCI validation dataset included data of third, fourth and fifth graders collected in 2010–2011 (*n* = 238 after removing data without valid responses for control items, 52.1% boys). For jTCI, data from a group of fifth graders (*n* = 101, 46.5% boys) collected by our research group in February 2019 (i.e., about 1 year before the COVID-19 pandemic outbreak) in schools of the same area (about 30 km away) in which data were collected for the present study in 2020. Fifth graders' jTCI normative data collected in 2010–2011 were thus compared with jTCI data collected in 2019 to verify if any change had occurred with time. Participant demographic characteristics of all these samples are detailed in Table 1.

**Table 1:** Characteristics of the samples used in the study.

	**Sample studied during the COVID-19 pandemic**	**Samples studied before the COVID-19 pandemic**		
	**PANAS-C, jTCI, FCV-19S (October-November 2020)**	**PANAS-C validation (2014)**	**jTCI validation (2010–2011)**	**jTCI unpublished (2019)**
*n*	323	331	238	101
Boys	180	171	124	47
Girls	143	160	114	54
Third graders	103	0	89	0
Fourth graders	75	112	77	0
Fifth graders	145	219	72	101

*PANAS-C, Positive and Negative Affect Scale for Children; jTCI, junior Temperament and Character Inventory; FCV-19S, Fear of Covid-19 Scale*.

Secondary analysis involved (ii) descriptive statistics for FCV-19S scores, Pearson's correlation of jTCI scores with PANAS-C and FCV-19S scores, and the comparison of PANAS-C and FCV-19S scores between low-resilience (LR) and high-resilience (HR) personality profiles groups of the 2020 dataset. LR and HR groups were obtained, as done in a previous work of our research group (85), by partitioning the whole sample on the basis of individual HA and SD scores from the jTCI questionnaire, since, as mentioned in the Introduction, these two scales have been reported as the most influential TCI scales on adults' self-reports of resilience (HA inversely and SD directly related to resilience) (71–73). The partitioning procedure was performed using the k-means algorithm (86) on the participants' standardized HA and SD scores. Comparison of PANAS-C and FCV-19S scores between LR and HR groups was performed using robust independent samples *t*-tests.

Sample size was determined by voluntary study participation in 2020 (*n* = 323) and by normative sample sizes of PANAS-C (*n* = 331) and jTCI (*n* = 238). In a statistical power analysis performed in terms of sensitivity, the sensitivity of study design for the primary analyses of our study was tested by comparing the effect sizes observed in the current study with the Minimum Detectable Effects (MDEs) obtained from the desired minimum statistical power of 0.80, an α level of 0.05, and the sample sizes employed in each pre- vs. during pandemic comparison. This power analysis revealed that the study design was generally sensitive enough to detect the differences of interest (in PANAS-C: for PA in girls *d* = 0.33, d_MDE_ = 0.37; in jTCI: for HA in boys *d* = 0.36, d_MDE_ = 0.33; for HA in boys and girls, *d* = 0.29, d_MDE_ = 0.24; for ST in boys and girls, *d* = 0.26, d_MDE_ = 0.24; effect sizes observed in the current study can be found in Tables 2, 3). For all tests, effects are reported as significant at *p* < 0.05.

**Table 2:** Comparison of Positive and Negative Affect Scale for Children (PANAS-C) data collected in Italian fourth and fifth graders before [2014, (75)] and during the COVID-19 pandemic (October-November 2020).

**Group**	**Scale**	**PANAS-C score before the pandemic (2014)**	**PANAS-C score during the pandemic (Oct-Nov 2020)**	***t* [95% CI]**	**p_**Bonf**_ (effect size)**
Boys	PA	42.0 ± 8.4	43.1 ± 6.5	−1.2 [−2.7;0.7]	0.46 (*d* = −0.14)
	NA	25.8 ± 7.6	24.7 ± 7.2	1.4 [−0.5;2.9]	0.34 (*d* = 0.16)
Girls	PA	43.0 ± 8.1	40.4 ± 7.5	2.5 [0.6;4.6]	0.02 * (d = 0.33)
	NA	27.4 ± 8.3	26.9 ± 7.9	0.4 [−1.6;2.5]	1.00 (*d* = 0.06)
Boys and Girls	PA	42.5 ± 8.3	42.0 ± 7.0	0.8 [−0.8;1.8]	0.87 (*d* = −0.10)
	NA	26.6 ± 8.0	25.6 ± 7.5	1.5 [−0.3;2.3]	0.27 (*d* = 0.12)

PA, Positive Affect; NA, Negative Affect. Asterisk indicates significant difference (*p < 0.05).

**Table 3:** Comparison of the junior Temperament and Character Inventory (jTCI) data collected in Italian third, fourth and fifth graders before [2010–2011, (77)] and during the COVID-19 pandemic (October-November 2020).

**Group**	**Scale**	**jTCI score before the pandemic (2010–2011)**	**jTCI score during the pandemic (Oct-Nov 2020)**	***t* [95% CI]**	**p_**Bonf**_ (effect size)**
Boys	NS	7.6 ± 3.1	7.1 ± 2.9	1.4 [−0.2;1.2]	1.00 (*d* = 0.17)
	HA	9.4 ± 4.5	7.9 ± 4.0	3.1 [0.6;2.5]	0.02* (*d* = 0.36)
	RD	4.2 ± 1.7	4.0 ± 1.9	1.1 [−0.2;0.6]	1.00 (*d* = 0.13)
	P	3.7 ± 1.4	3.9 ± 1.3	−1.1 [−0.5;0.1]	1.00 (*d* = −0.13)
	SD	11.6 ± 3.0	12.4 ± 3.1	−2.2 [−1.5;−0.1]	0.19 (*d* = −0.26)
	C	14.0 ± 3.0	14.3 ± 3.1	−0.9 [−1.0;0.4]	1.00 (*d* = −0.11)
	ST	5.2 ± 2.1	5.6 ± 2.0	−2.1 [−1.0;0.0]	0.27 (*d* = −0.24)
Girls	NS	6.3 ± 3.1	5.5 ± 2.8	2.0 [0.0;1.5]	0.31 (*d* = 0.25)
	HA	10.3 ± 4.0	9.6 ± 4.1	1.4 [−0.3;1.7]	1.00 (*d* = 0.17)
	RD	5.0 ± 1.6	4.9 ± 1.9	0.4 [−0.3;0.5]	1.00 (*d* = 0.05)
	P	3.7 ± 1.4	4.0 ± 1.3	−1.8 [−0.6;0.0]	0.48 (*d* = −0.23)
	SD	12.5 ± 3.7	12.5 ± 3.5	−0.1 [−0.9;0.9]	1.00 (*d* = −0.01)
	C	15.1 ± 2.3	15.8 ± 2.3	−2.3 [−1.2;−0.1]	0.14 (*d* = −0.29)
	ST	5.3 ± 2.1	5.9 ± 1.9	−2.2 [−1.1;−0.1]	0.18 (*d* = −0.28)
Boys and Girls	NS	6.9 ± 3.2	6.4 ± 3.0	2.2 [0.1;1.1]	0.21 (*d* = 0.19)
	HA	9.9 ± 4.3	8.7 ± 4.1	3.3 [0.5;1.9]	<0.01* (*d* = 0.29)
	RD	4.6 ± 1.7	4.4 ± 1.9	1.3 [−0.1;0.5]	1.00 (*d* = 0.11)
	P	3.7 ± 1.4	4.0 ± 1.3	−2.0 [−0.5;0.0]	0.29 (*d* = −0.17)
	SD	12.0 ± 3.4	12.4 ± 3.3	−1.5 [−1.0;0.1]	1.00 (*d* = −0.13)
	C	14.5 ± 2.7	15.0 ± 2.9	−1.9 [−0.9;0.0]	0.44 (*d* = −0.16)
	ST	5.2 ± 2.1	5.8 ± 2.0	−3.0 [−0.9;−0.2]	0.02* (*d* = −0.26)

NS, Novelty Seeking; HA, Harm Avoidance; RD, Reward Dependence; P, Persistence; SD, Self-Directedness; C, Cooperativeness; ST, Self-Transcendence. Asterisk indicates significant difference (*p < 0.05).

## Results

### Affect

**Figure 1:**
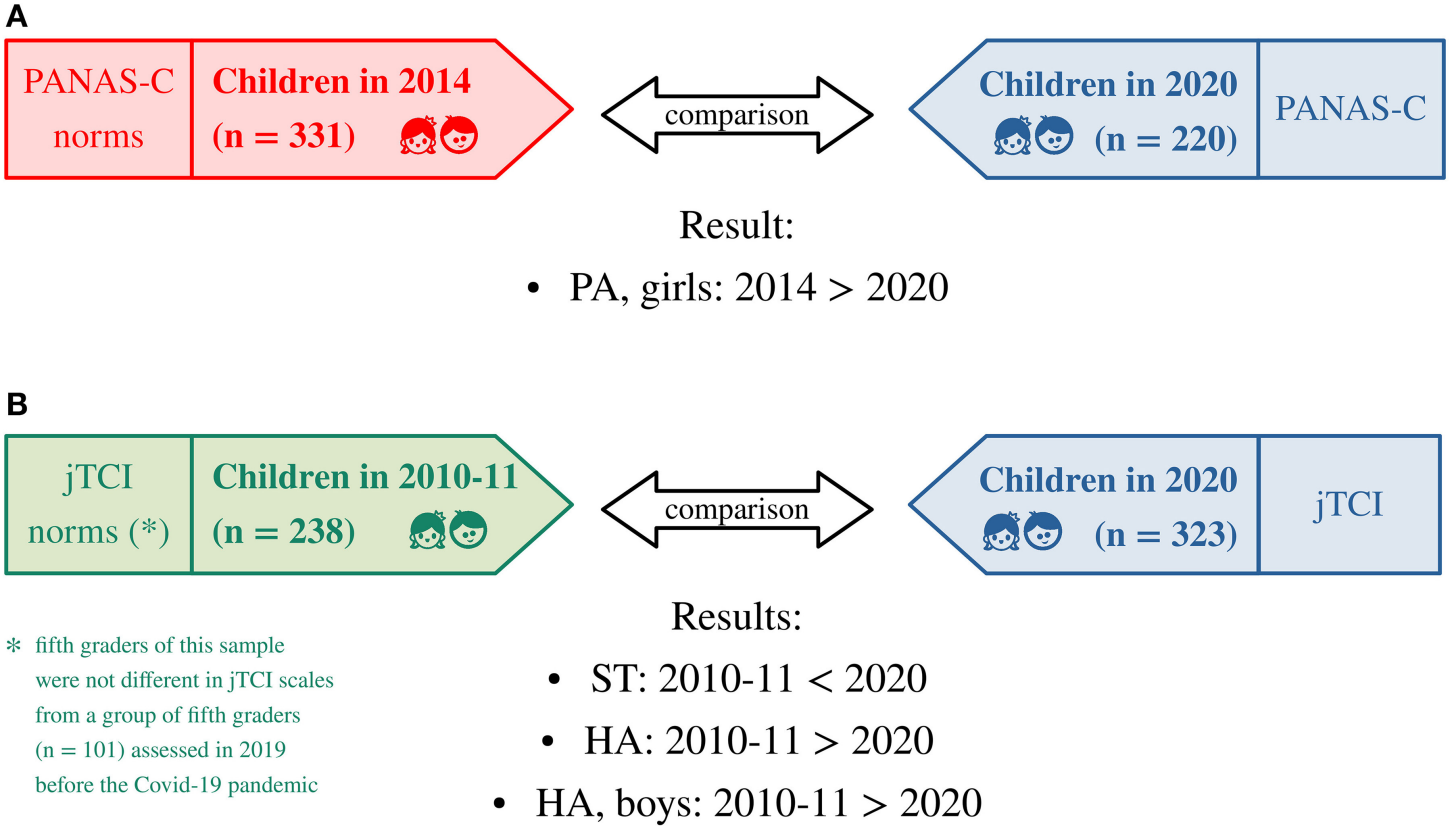
Primary analyses of the study. **(A)** Comparison of PANAS-C scores collected during the pandemic with normative PANAS-C scores collected in 2014 (75) (the normative sample included fourth and fifth graders' data and, therefore, only data of fourth and fifth graders assessed in 2020 were included in the analysis). **(B)** Comparison of jTCI scores collected during the pandemic with normative jTCI scores collected in 2010–11 (77). PANAS-C, Positive And Negative Affect Scale for Children; PA, Positive Affect; jTCI, junior Temperament and Character Inventory; HA, Harm Avoidance; ST, Self-Transcendence.

The comparison of fourth and fifth graders' data collected before the pandemic (*n* = 331, 51.7% boys) with fourth and fifth graders' data collected in 2020 (*n* = 220, 59.1% boys) generally showed no differences in positive and negative affect, except for a difference in girls' positive affect [*t*(198.5) = 2.5, p_Bonf_ = 0.02]: in 2020 girls self-reported a significantly lower positive affect than girls in 2014 (see Table 2, Figure 1A).

### Personality

The comparison of jTCI data collected before the pandemic in 2010–2011 (*n* = 238, 52.1% boys) with data collected in 2020 (*n* = 323, 55.7% boys) showed a significant difference in HA scores in boys [M_2010−2011_ > M_2020_, *t*(245.2) = 3.1, p_Bonf_ = 0.02] and in the full sample of boys and girls [M_2010−2011_ > M_2020_, *t*(501.0) = 3.3, p_Bonf_ = 0.006]. A significant difference in ST scores [M_2010−2011_ < M_2020_, *t*(494.9) = −3.0, p_Bonf_ = 0.02] was also observed in the full sample of boys and girls. Children assessed during the pandemic showed lower HA and higher ST scores than children assessed before the pandemic in 2010–2011 (see Table 3, Figure 1B). It is worth noting that no difference was observed between fifth graders' jTCI data collected in 2010–2011 and fifth graders' jTCI data collected in 2019 (for all scales, in boys/girls/boys and girls: |t| < 2.4, p_Bonf_ > 0.12).

### Fear of COVID-19

**Figure 2:**
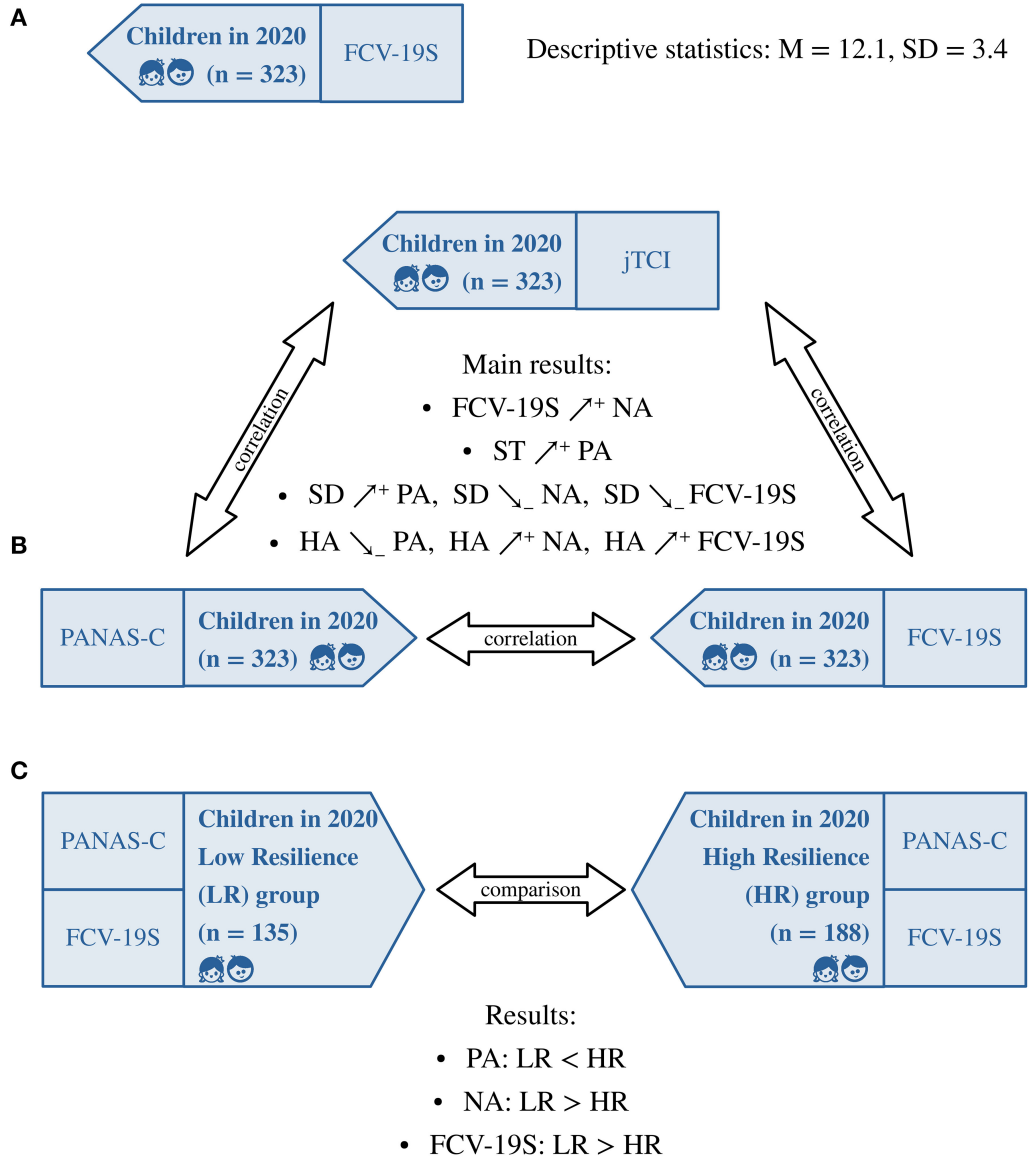
Secondary analyses of the study. **(A)** Descriptive statistics of FCV-19S scores. **(B)** Correlation of PANAS-C, FCV-19S and jTCI scores collected during the pandemic. **(C)** Comparison of PANAS-C and FCV-19S scores collected during the pandemic between a Low-Resilience and a High-Resilience personality profile group (the two groups were obtained partitioning the whole study sample on the basis of individuals' HA and SD scores from the jTCI questionnaire). PANAS-C, Positive And Negative Affect Scale for Children; PA, Positive Affect; NA, Negative Affect; FCV-19S, Fear of COVID-19 Scale; jTCI, junior Temperament and Character Inventory; HA, Harm Avoidance; SD, Self-Directedness; ST, Self-Transcendence; ↗^+^, positive correlation; ↘_−_, negative correlation.

There were extremely few missing values (0.25% of the total number of responses). Participants' average score (boys: 11.6 ± 3.4, girls: 12.7 ± 3.2, boys and girls: 12.1 ± 3.4, see Table 4, Figure 2A) was close to that obtained in a sample of 340 girls during the second wave of COVID-19 in Iran (M = 12.1 for third graders, M = 12.8 for fourth graders, M = 10.6 for fifth graders; data collected from July to November 2020, *n* = 340, 100% girls, age: 10.1 ± 1.7 years) (38), but lower than scores obtained during the first wave of COVID-19: in Canadian children the average FCV-19S score was 14.1 ± 5.7 (data collected between April and May 2020, *n* = 144, 51.4% boys, age: 9 to 12 years) (36), in Turkish children/adolescents was 18.9 ± 6.3 (data collected from April to June 2020, *n* = 381, 50.4% males, age: 15.4 ± 2.4 years) (37) and in Italian adults was 16.9 ± 6.1 (data collected from 18 March to 21 March 2020, *n* = 249, 8.0% men, age: 34.5 ± 12.2 years) (76).

**Table 4:** Descriptive statistics of the Fear of COVID-19 Scale (FCV-19S) scores collected during the pandemic (October-November 2020).

**Group**	**FCV-19S total score**
	**(Oct-Nov 2020)**
Boys	11.6 ± 3.4
Girls	12.7 ± 3.2
Boys and Girls	12.1 ± 3.4

### Correlations

Table 5 depicts the correlation matrix of PANAS-C, FCV-19S and jTCI measures. The exploration of the relationship between affectivity and personality showed that: positive affect (PA) was positively correlated with P, SD and ST, as well as negatively correlated with HA; negative affect (NA) was positively correlated with NS and HA, as well as negatively correlated with P and SD. The exploration of the relationship between fear of COVID-19 and personality showed that fear was positively correlated with HA and negatively correlated with SD. Moreover, correlation analysis showed a positive relationship between negative affect and fear of COVID-19 (see also Figure 2B).

**Table 5:** Correlation matrix of the PANAS-C, FCV-19S and jTCI scores obtained during the COVID-19 pandemic (October-Novembre 2020).

**Measure**	**1**.	**2**.	**3**.	**4**.	**5**.	**6**.	**7**.	**8**.	**9**.
1. PANAS–C PA									
2. PANAS–C NA	−0.24**								
3. FCV.19S	0.01	0.25***							
4. jTCI NS	−0.04	0.15*	−0.08						
5. jTCI HA	−0.23***	0.35***	0.28***	−0.03					
6. jTCI RD	0.15	−0.07	−0.02	−0.27***	−0.01				
7. jTCI P	0.15**	−0.16*	0.04	−0.24***	−0.22***	0.19***			
8. jTCI SD	0.16*	−0.26***	−0.17**	−0.27***	−0.32***	0.21***	0.38***		
9. jTCI C	0.15	−0.06	0.07	−0.42***	−0.03	0.24***	0.28***	0.32***	
10. jTCI ST	0.13*	0.06	0.11	−0.06	0.09	0.07	0.08	−0.12	0.24***

PANAS-C, Positive and Negative Affect Scale for Children; PA, Positive Affect; NA, Negative Affect; FCV-19S, Fear of COVID-19 Scale; jTCI, junior Temperament and Character Inventory; NS, Novelty Seeking; HA, Harm Avoidance; RD, Reward Dependence; P, Persistence; SD, Self-Directedness; C, Cooperativeness; ST, Self-Transcendence. Asterisks indicate significant correlations (*p < 0.05, **p < 0.01, ***p < 0.001).

#### Low and High Resilience Profile Groups

Based on individuals' standardized HA and SD scores, the whole group of children assessed in 2020 was partitioned in a low-resilience (LR; *n* = 135, 51.9% boys) and a high-resilience (HR; *n* = 188, 58.5% boys) group. In comparison with children in the HR group (see Table 6, Figure 2C), children in the LR group had significantly lower PA scores [*t*(158.5) = 2.5, *p* = 0.01], higher NA scores [*t*(146.9) = −5.4, *p* < 0.0001] and higher FCV-19S scores [*t*(185.2) = −4.9, *p* < 0.0001]. No difference between the two groups was observed in term of gender composition [χ^2^ (1, *N* = 323) = 1.2, *p* = 0.28].

**Table 6:** Differences in the low-resilience (LR) and high-resilience (HR) profile groups.

**Questionnaire**	**Measure**	**Group**		***t* [95% CI]**	***p* (effect size)**
		**LR**	**HR**		
PANAS-C	PA	40.9 (7.5)	43.0 (6.9)	2.5 [0.4;3.8]	0.01* (*d* = 0.28)
	NA	28.6 (8.8)	23.4 (6.8)	−5.4 [−7.0;−3.2]	<0.0001*** (*d* = −0.60)
FCV-19S	Total score	13.2 (3.6)	11.3 (2.9)	−4.9 [−3.0;−1.3]	<0.0001*** (*d* = −0.55)

PANAS-C, Positive and Negative Affect Scale for Children; PA, Positive Affect; NA, Negative Affect; FCV-19S, Fear of COVID-19 Scale (*p < 0.05, ***p < 0.001).

## Discussion

This study investigated primary school students' self-reports during the second wave of COVID-19 in Italy. Three questionnaires were used, one for assessing students' temperament and character dimensions of personality (jTCI), one for positive and negative affect (PANAS-C), and one for fear of COVID-19 (FCV-19S). Data analysis focused on: (i) comparing the affect and personality scores obtained during the pandemic with same-graders' scores obtained before the pandemic (during the validation of the affect and personality questionnaires in Italy); in the data collected during the pandemic (ii) describing the distribution of fear of COVID-19 scores, correlating affect and fear of COVID-19 with personality scores, and comparing affect and fear of COVID-19 scores between a low-resilience and a high-resilience profile group.

In the pre- vs. during pandemic comparison of affect scores, no differences were found in terms of positive and negative affect in the overall sample (boys and girls). A significant difference between data collected before and during the pandemic, however, was found in girls' positive affect: in 2020 girls self-reported a significantly lower positive affect than girls in 2014. There are few studies that collected primary school children's self-reports during the COVID-19 pandemic and that could compare their data with those collected prior to the pandemic (30, 87–90). The only existing study that carried out this comparison using children's self-reports of affectivity (30) found out that positive or negative affect scores collected in 34 healthy children (age: 11.9 ± 1.2 years) by using the shortened 10-item PANAS-C in California from 22 April to 29 July 2020 did not differ from data collected in other pediatric studies conducted prior to the pandemic (*n* = 101); nonetheless, the same children assessed in that study during 2020 reported significantly greater state anxiety (measured with the State Anxiety Inventory for Children) (91) compared to children assessed prior to the pandemic. It is therefore possible that measurements of children's affectivity, such as PANAS-C, could not capture the psychological impact of the COVID-19 pandemic on children that has instead been reported in other pre- vs. during pandemic studies in terms of anxiety (87, 88), depression or post-traumatic symptoms (89). Two of these three studies (87, 88), however, included samples of children and adolescents up to 17 years without distinguishing children from adolescents in the analyses, when various studies [e.g., (92, 93)] and reviews (12, 16) reported greater severity of anxiety, depression and stress symptoms in adolescents than in primary school children during 2020. It is worth noting that a study on 166 fourth graders (84 boys and 82 girls) carried out in Korea in September and October 2020 (90) detected unchanged levels of life satisfaction, measured with the Satisfaction with Life Scale (94), with respect to data collected in 2018 and 2019.

In the pre- vs. during pandemic comparison of personality scores performed in the present study, a significant change was observed in the overall sample in harm avoidance (decreased in 2020) and self-transcendence (increased in 2020) scores. In the biopsychosocial model of personality, on which the Temperament and Character Inventory is based, harm avoidance is the dimension of temperament linked to worry/pessimism, fear of uncertainty, shyness and fatigability (64, 77, 95). Although temperament should bear a greater stability throughout life compared to character (96), among the temperamental traits harm avoidance is considered to be the most susceptible to mood and anxiety (97, 98), as well as to experiences such as trainings [e.g., (99, 100)] or therapy [e.g., (101, 102)]. In our study, a decreased level of harm avoidance in the overall sample was observed in comparison with pre-pandemic data, which was mainly due to the decrease of scores in boys. This means that in this dimension of temperament, children self-reported in 2020 a generally healthier profile than children assessed in 2010–2011. This result seems to be in contrast with the increase of children's anxiety and depression symptoms which were generally reported, although not consistently [e.g., Ravens-Sieberer et al. (88) observed no significant increase in the prevalence of depressive symptoms before vs. during the pandemic], in the previous literature focusing on the pandemic period. When comparing the results of the various studies on the impact of the pandemic, an important issue concerns when and where these studies were carried out, because the environmental conditions during the different phases/waves of the pandemic could have differently influenced the mental condition of people that were exposed to them: for example, children in our study were experiencing the second wave of COVID-19 in Italy, but were back to school in September 2020 after their schools had remained closed since the outbreak of the pandemic in February 2020, and could therefore find themselves in a different condition than their German or Swedish peers who returned to school in May 2020 or who did not experience school closures (103). That being said, the change in Italian children's harm avoidance may look like a positive rebound in terms of optimism, courage and energy after the possibly traumatic experience of the first wave of COVID-19 and the hard lockdown imposed in Italy. The fact that this change remained within one standard deviation from normative scores suggests, however, that children's personality did not change dramatically from pre-pandemic levels and, in particular, toward excessive and unhealthy fearlessness and imprudence profiles.

The observed decrease in harm avoidance scores from pre-pandemic levels was accompanied by the increase from pre-pandemic levels in the character trait of self-transcendence, although no correlation was found between these two variables. In the biopsychosocial model of personality, self- transcendence is the dimension of character linked to fantasy/daydreaming, transpersonal identification and spiritual acceptance (64, 77, 95). Changes in adults' self-transcendence have repeatedly been observed in response to trainings/therapy and medical treatment (104–108). Self-transcendence and spirituality are recognized as useful coping strategies for managing stressful life events (109, 110) and it is therefore possible that children in our study drew on their spiritual resources in response to the pandemic crisis for developing resilience. This possibility can be encompassed within the concept of post-traumatic growth, defined as “positive change experienced as a result of the struggle with trauma” (111), one of whose domains being precisely spiritual change: various meta-analytic studies revealed that post-traumatic growth is in general positively associated with spirituality in adults and children (112–115). During the COVID-19 emergency, large portions of the population had to simultaneously confront, directly or not, confinement, illness and death. Such an experience can have stimulated the development of spirituality/self-transcendence, understood as the discovery or making sense of the experience itself: this healing process can pass through an initial crisis, as reported for example in a study on adults during the first days of the COVID-19 lockdown in Italy, where 1,250 adults self-reported significantly worse levels of mental health and lower levels of spiritual well-being in comparison with pre-pandemic normative data (116). As seen for harm avoidance, the change in self-transcendence observed in our study also remained within one standard deviation from normative levels, which can be interpreted as a significant but not dramatic modification of character maturity (at the transpersonal level).

In our study, children's fear of COVID-19 was also assessed and, despite the paucity of other observations of this measure in children, the participants' average score seemed to be similar to that obtained by other studies during the second wave of COVID-19 (in Iranian girls) and lower than those obtained during the first wave (in Canadian children, Turkish children/adolescents and in Italian adults). A significant decrease from the first to the second wave in the fear of COVID-19 scores (assessed with the same scale used in our study) has been observed, for example, in adult Slovakians (34). This can be viewed as the result of the individual and institutional adaptation to the pandemic after the initial emergency response. Importantly, in our study children's fear of COVID-19 scores resulted to be positively correlated with harm avoidance scores and negatively correlated with self-directedness scores. As already mentioned, these two scales have been reported as the most influential Temperament and Character Inventory scales on adults' self-reports of resilience [harm avoidance negatively and self-directedness positively related to resilience, (71–73)] and thus, in the present study, children with a weaker resilience profile self-reported higher fear of COVID-19 scores than children with a stronger resilience profile. In the analysis of the two resilience profile groups, it was also observed that children in the low resilience profile group self-reported significantly higher negative affectivity and lower positive affectivity than children in the high resilience profile group.

Other salient results coming from the correlations between the study variables were: harm avoidance directly related to negative affect and inversely related to positive affect; persistence [the temperament trait linked to determination to achieve a goal despite frustration or fatigue, (64, 77, 95)] and self-directedness directly related to positive affect and inversely to negative affect; self-transcendence directly related to positive affect. These results seem to confirm that children with lower personality tendency toward worry/pessimism, fear of uncertainty, shyness and fatigability (trait of harm avoidance) and higher personality tendency to self-identification as an integral part of the universe as a whole (trait of self-transcendence) were likely to live with more positive and less negative feelings than children with the opposite features of personality. Results indicate also that the same condition of experiencing more positive and less negative feelings was also related to personality traits of maturity, autonomy and reliability (trait of self-directedness), as well as of diligence and determination (trait of persistence).

The present study has some strengths, in comparison with similar studies, as well as several limitations. The strengths include (i) the fact that children's self-reports, rather than proxy reports, were used and (ii) that these self-reports were obtained in classroom, rather than online. The limitations include that (i) pre- vs. during pandemic differences in the study measures have been related exclusively to the pandemic, whereas other individual and contextual factors may have influenced these differences; (ii) differently from jTCI (for which the normative dataset and a dataset collected in 2019 were used as pre-pandemic datasets), for PANAS-C it was not possible to obtain a dataset collected immediately before the pandemic, that confirmed the normative dataset collected in 2014; (iii) pre- vs. during pandemic differences in the study measures were observed using different groups of children, which seems the best way to assess an average change in a population (comparing it with a normative sample), but, at the same time, due to the fact that assessment is performed at group levels, cannot take into account individual longitudinal changes in single children; (iv) the FCV-19S is a tool developed and validated for adults, although children in our study filled it easily (very few missing responses) and results seemed to be consistent with those obtained using the other study measures; (v) results were obtained in Italy immediately before the peak of the second wave of infections of COVID-19 and it is not possible to know to what extent these results can be generalizable to other periods and countries, as previously discussed.

In conclusion, our findings suggest that Italian primary school children, exposed to the first wave of COVID-19 and the hard lockdown imposed in Italy during spring 2020, and assessed during the ascending phase of the second wave of the pandemic in Italy, had affect scores generally in line with normative data collected prior to the pandemic and personality profiles denoting increased levels of courage/optimism and spirituality in comparison with the typical, pre-pandemic, profiles of children's personality.

## Data Availability Statement

The raw data supporting the conclusions of this article will be made available by the authors upon request.

## Ethics Statement

The studies involving human participants were reviewed and approved by Local Ethics of the University of Udine, DILL, University of Udine. Written informed consent to participate in this study was provided by the participants' legal guardian/next of kin.

## Author Contributions

FF and CC: conceptualization. AM, FF, AP, and CC: methodology. AM: software, writing—original draft preparation, and visualization. AM and CC: validation and formal analysis. AM and AP: investigation. FF and CC: resources, supervision, and funding acquisition. AM, CU, AB, and EC: data curation. CU, EC, CC, and AB: writing—review and editing. AM, AP, and CC: project administration. All authors have read and agreed to the published version of the manuscript.

## Funding

This research was funded by the Prevention Department of Regione Autonoma Friuli-Venezia Giulia.

## Conflict of Interest

The authors declare that the research was conducted in the absence of any commercial or financial relationships that could be construed as a potential conflict of interest.

## Publisher's Note

All claims expressed in this article are solely those of the authors and do not necessarily represent those of their affiliated organizations, or those of the publisher, the editors and the reviewers. Any product that may be evaluated in this article, or claim that may be made by its manufacturer, is not guaranteed or endorsed by the publisher.
